# Magnetic Resonance Accuracy in the Diagnosis of Anterior Talo-Fibular Ligament Acute Injury: A Systematic Review and Meta-Analysis

**DOI:** 10.3390/diagnostics11101782

**Published:** 2021-09-28

**Authors:** Michela Barini, Domenico Zagaria, Davide Licandro, Sergio Pansini, Chiara Airoldi, Massimiliano Leigheb, Alessandro Carriero

**Affiliations:** 1 Department of Radiodiagnostic and Interventional Radiology, AOU Maggiore della Carità, 28100 Novara, Italy; barinimichela@gmail.com (M.B.); 20004369@studenti.uniupo.it (D.L.); 20032855@studenti.uniupo.it (S.P.); profcarriero@virgilio.it (A.C.); 2 Unit of Medical Statistics and Epidemiology, Department of Translation Medicine, University of Piemonte Orientale, AOU Maggiore della Carità, 28100 Novara, Italy; chiara.airoldi@uniupo.it; 3 Orthopaedics and Traumatology Unit, Department of Health Sciences, University of Piemonte Orientale, AOU Maggiore della Carità, 28100 Novara, Italy; massimiliano.leigheb@uniupo.it

**Keywords:** acute ATFL injury, lateral ankle trauma, diagnostic accuracy, MRI accuracy, systematic review, meta-analysis

## Abstract

Background: The studies about injury to the anterior talo-fibular ligament (ATFL) are focused mainly on chronic symptoms and chronic instability, and the literature about the accuracy of magnetic resonance imaging (MRI) in acute injuries is quite lacking. Methods: This systematic review with meta-analysis analyzes the diagnostic accuracy of MRI on acute ATFL injury. Relative studies were retrieved after searching three databases (MEDLINE, SCOPUS, and Cochrane Central Register of Controlled Trails). Eligible studies were summarized. The quality of the included articles was assessed using the revised Quality Assessment of Diagnostic Accuracy Studies (QUADAS-2) tool. Data were extracted to calculate pooled sensitivity and specificity of MRI. Results: Seven studies met our inclusion and exclusion criteria. For MRI, the pooled sensitivities and specificity in diagnosing acute ATFL injury were respectively 1.0 (95% CI: 0.58–1) and 0.9 (95% CI: 0.79–0.96). Pooled LR+ and LR− were respectively 10.4 (95% CI: 4.6–23) and 0 (95% CI: 0–0.82). Conclusion: This systematic review with meta-analysis investigated the accuracy of imaging for the diagnosis of acute ATFL injury. Our results demonstrated that MRI shows high diagnostic accuracy in the diagnosis of acute ATFL lesions. These results suggest that routine MRI in the case of suspected ATFL acute injury may be clinically useful, although this is not done in clinical practice due probably to high cost.

## 1. Introduction

Ankle sprains are among the most common injuries during sport events, accounting for up to 40% of all athletic injuries [1]. The lateral ankle ligament complex consists of the anterior talofibular ligament (ATFL), the calcaneo-fibular ligament (CFL) and the posterior talofibular ligaments (PTFL). The ATFL has the main role in resisting inversion in plantarflexion and anterolateral translation of the talus in the mortise. Considering both isolated and combined ligament injury, the ATFL can be damaged in up to 90% of major ankle injuries, compared to the CFL in 50–75% and PTFL in only 10% [2,3,4].

The most common mechanism of injury in lateral ankle sprain occurs with an excessive inversion and internal rotation of the rearfoot coupled with external rotation of the lower leg, which result in strain to the lateral ligaments. If the strain in any of the ligaments exceeds their tensile strength, ligamentous damage occurs. The initial grade of plantar flexion appears to be correlated with the likelihood of suffering a lateral ankle ligament lesion [5,6].

The early physical examination is based on observation of any gross dislocation or asymmetry. Palpation of ankle ligaments and evaluation for tenderness, including the medial ankle and length of the fibula, should be conducted. Visually apparent edema and ecchymosis should be evaluated, and the range of motion of the affected ankle joint compared with the contralateral ankle, muscular strength and neurovascular status [7,8,9].

Magnetic resonance imaging (MRI), ultrasonography (US), stress radiography, and arthrography can be utilized for diagnosing chronic lateral ankle ligament injury [10].

Conventional stress-radiography is basically helpful to exclude fractures, even though the frequency of this complication occurs in less than 15% of ankle sprains [11].

Although ultrasonography (US) has been found to be an effective method for evaluating the integrity of the ankle ligaments, it has been shown to have an accuracy rate of 91% in the diagnosis of ATFL injuries versus 97% for MRI [12,13]; moreover, MRI is superior in evaluations of bone marrow and articular cartilage abnormalities, which are also important clinical issues in acute settings [13].

The accuracy of the routine ankle MRI protocol for diagnosis of acute ATFL injury is still in doubt because of its wide range of specificity (70–97%) and sensitivity (40–95%) [14,15,16,17]. MRI may show detachment, discontinuity, thickening, thinning, contour irregularity of the ligament, a bright rim sign [16] or an associated bony avulsion [18,19,20,21]. Both US and MRI were equally sensitive in detecting the presence (or absence) of injury to the ankle muscle, tendons and ligaments, though US was less specific than MRI in detecting grade III injury [22]. Arthroscopic or surgical findings are considered the gold standards for ligament injuries [17,23,24].

After conservative or surgical treatment, 10 to 30% of patients have chronic symptoms, including persistent synovitis or tendinitis, ankle stiffness, swelling, pain, muscle weakness and ‘giving way’ [25]. Well-designed physical therapy programmes usually reduce instability. For individuals with chronic instability refractory to conservative measures, surgery may be needed [24,25].

Few studies evaluated the acute injury of lateral ankle ligamentous complex, thus different sensitivities and specificities for detecting ATFL tears have been reported. These studies have focused mainly on chronic symptoms and chronic instability, and the literature is quite lacking about the accuracy of MRI in acute injuries. The variability makes it difficult to justify its usage in identifying lateral ligamentous injuries due to the prohibitive costs of MRI.

Nevertheless, MRI was found to be able to accurately diagnose lateral ankle ligament tears in most cases.

## 2. Materials and Methods

### 2.1. Inclusion and Exclusion Criteria

The study was reported according to the PRISMA guideline [26].

The following criteria were used to include qualified studies: (1) cohort-type or cross-sectional studies; (2) evaluated MRI for the diagnosis of acute ATFL, with MRI performed within three months of the injury; (3) compared imaging results with arthroscopic or surgical findings as reference standards; and (4) reported data that enabled the calculation of the respective numbers of true positive (TP), true negative (TN), false positive (FP), and false negative (FN).

The studies that met the following criteria were excluded: (1) chronic injury patients; (2) patients with confounding factors like ankle fracture or a history of previous foot and/or ankle surgeries; (3) did not clearly describe arthroscopic or surgical findings as their reference standards; (4) cadaveric studies or studies utilizing animal models; and (5) non-English articles.

### 2.2. Search Strategy

We conducted a systematic review of the literature on the following three databases: MEDLINE, SCOPUS, and Cochrane Central Register of Controlled Trials (CENTRAL) and reported findings according to PRISMA guidelines [26]. The detailed search strategies were first developed in MEDLINE and were then adjusted and applied to the other two databases (Table 1).

Selected articles from each database were first screened for duplication. Then, a screening of titles and abstracts was conducted, and studies relevant to this systematic review underwent full-text selection. Qualified studies were included according to the inclusion and exclusion criteria mentioned above.

### 2.3. Data Extraction and Quality Assessment

Extracted data included authors, year of publication, participant demographics, study design, index test, gold standard, and the number of true positive, false negative, false positive, and true negative subjects.

The pathological features of acute injury refer to various manifestations on the images [27,28,29], referring to different types of injuries defined as “stretching”, “rupture”, “scarring” or “thickening”, all of which are classified as “injured”. We have eliminated this diversity by dichotomizing the results of the images as “injured” and “intact” for adequate comparability between the different studies included.

Two authors independently extracted this data and compiled a custom checklist for this review. The results of the two authors were cross-validated and the discrepancies were mediated by the third author. The quality of the included articles was assessed using the revised Quality Assessment of Diagnostic Accuracy Studies (QUADAS-2) tool, through which the risk of bias was assessed in terms of patient selection, index test and reference standard [30].

### 2.4. Statistical Analysis

Pooled estimates of sensitivity, specificity, and positive/negative likelihood (with corresponding 95% confidence intervals [CIs]) were analyzed based on the bivariate model [31]. The pooled diagnostic odds ratio (DOR) was not reported because individual DOR could only be determined in one study and this is not unexpected in the case of a few studies and many empty cells [32].

To graphically present the results and to facilitate the visualization of the threshold effect, we plotted the summary receiver operating characteristic (SROC) curve and the forest plots. The heterogeneity was evaluated with the Cochran’s Q and Higgins’ I2 statistics [33].

To obtain the post-test probability, we also reported Fagan’s nomogram [34]. As a concern for meta-analysis of diagnostic trials, publication bias was tested using the funnel plot and Deeks test [35].

All analyses were performed with the command “MIDAS”? [36] belonging to the STATA package.

## 3. Results

### 3.1. Description of the Included Studies

**Figure 1:**
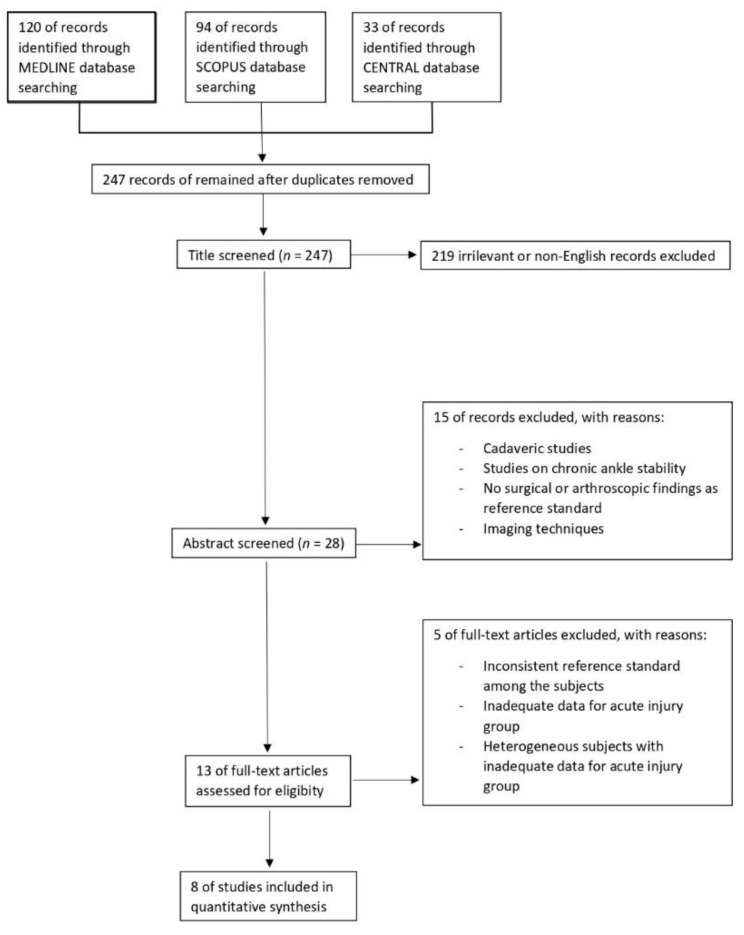
Flow diagram of search strategy.

A total of 120 articles were retrieved from MEDLINE, 94 articles were retrieved from SCOPUS, and 33 articles were retrieved from CENTRAL. After eliminating duplicate articles, a total of 247 studies were identified in the primary search of the three databases above (Figure 1).

Subsequently, 219 studies were excluded as irrelevant or non-English records. The remaining 28 studies were abstract screened, and consequently 15 articles were deleted for the following reasons: studies on cadavers, no surgical or arthroscopic findings as reference standard, and other imaging techniques.

After this screening, 13 studies were selected for a full-text evaluation; of these, five were excluded for the following reasons: inconsistent reference standard among the subjects, inadequate data for acute injury group, heterogeneous subjects with inadequate data for acute injury group. Table 2 shows the characteristics of the eight studies included in the final analysis, both for the selected patients and for the MRI protocol.

Table 3 reports the methodological qualitative assessment using the QUADAS2 tools.

### 3.2. Results of Meta-Analysis

The data were available in eight studies [37,38,39,40,41,42,43,44], but we only analyzed seven because one (Kreitner 1998) did not recruit any non-diseased subjects.

**Figure 2:**
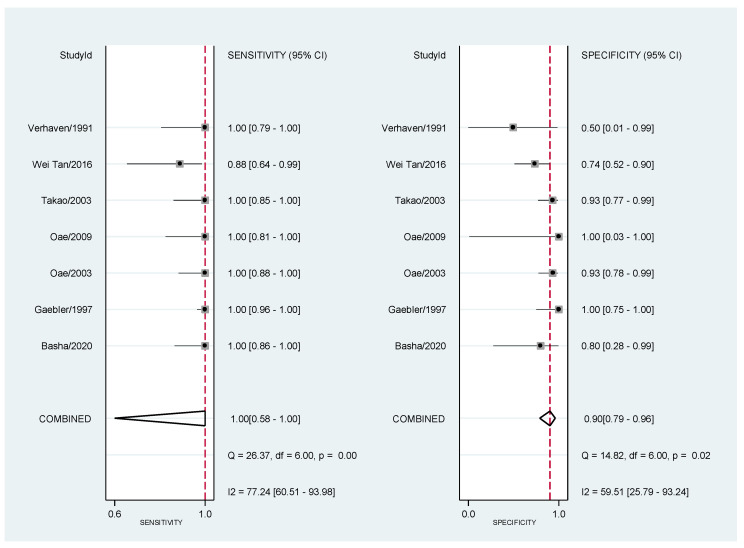
Forest plot of Magnetic Resonance Accuracy in the diagnosis of anterior talo-fibular ligaments acute injury. Note: we reported sensitivity and specificity of each study included in the analysis.

Sensitivity and specificity of the individual studies are shown in Table 4 and Figure 2.

The heterogeneity tests, Cochran’s Q and Higgins I2, were Q = 2.98 P = 0.112 and I2 = 33%. Both of these tests indicate modest heterogeneity between studies.

**Figure 3:**
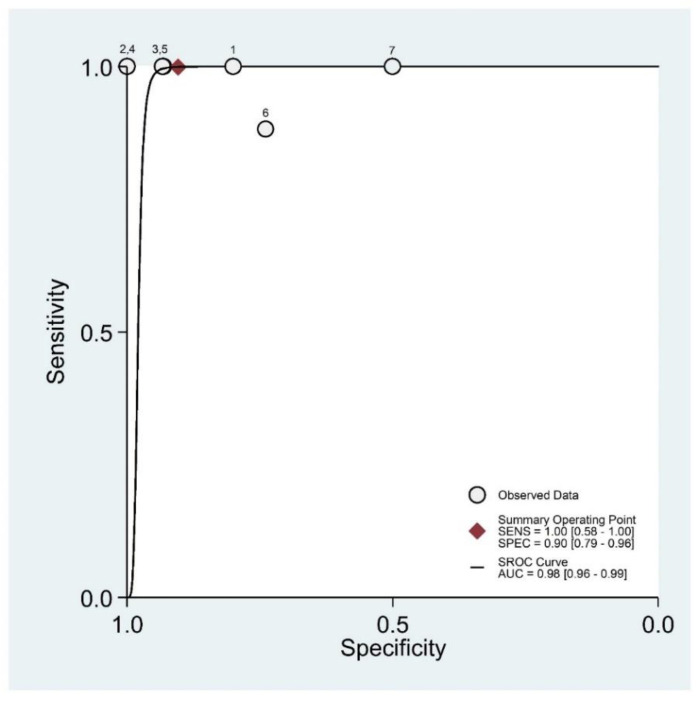
Summary Receiver Operating Characteristic (SROC) curve for Magnetic Resonance Accuracy in the diagnosis of anterior talo-fibular ligaments acute injury.

The area under the SROC curve (AUC) was 0.98 (95% CI: 0.96–0.99) (Figure 3).

**Figure 4:**
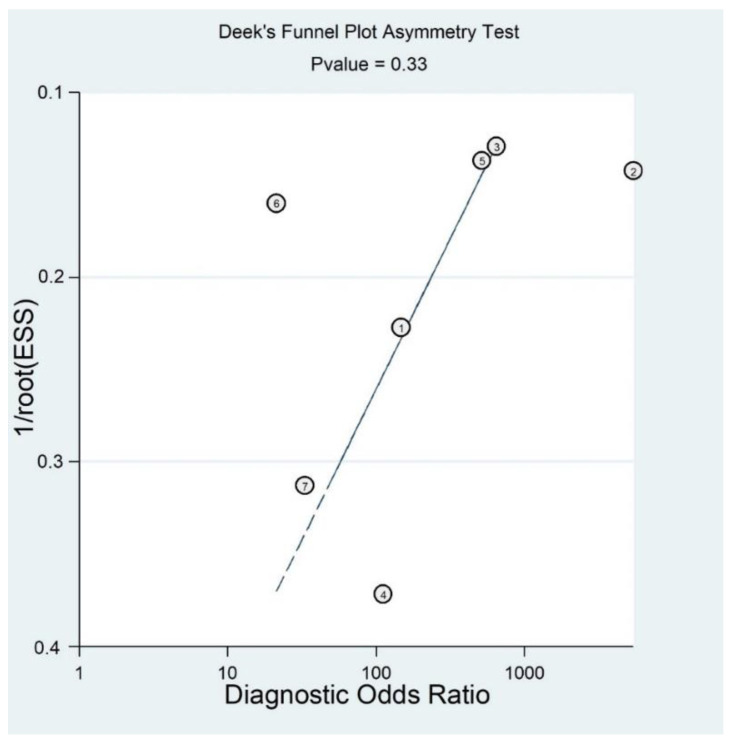
Funnel plot for Magnetic Resonance Accuracy in the diagnosis of anterior talo-fibular ligaments acute injury.

In Figure 4 we presented a funnel plot to evaluate the presence of publication bias, though it is of questionable validity in the context of diagnostic test accuracy meta-analysis [32,42].

As a result, the funnel plot seemed symmetrical with a *p* value of 0.33, and this suggested a low risk of publication bias.

**Figure 5:**
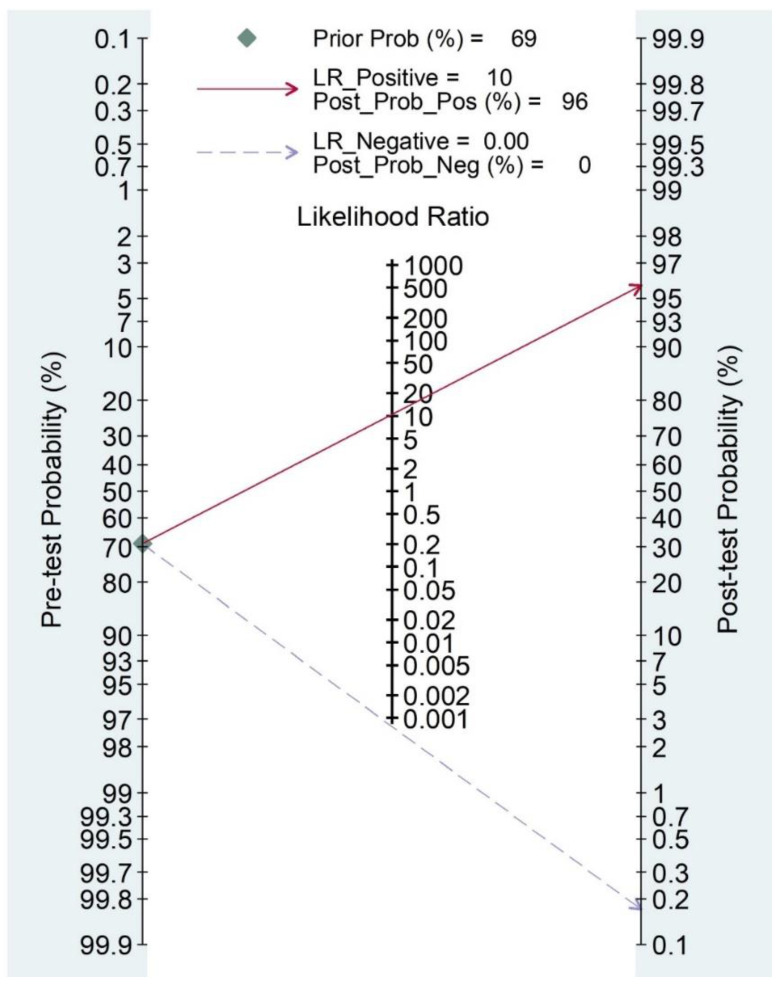
Fagan’s nonogram for the calculation of post-test probabilities.

To evaluate the post-test probability, we reported Fagan’s nomogram (Figure 5) that had a prevalence of 69% of anterior talo-fibular ligaments acute injury.

A pre-test probability of 69% for an anterior talo-fibular ligaments acute injury was fixed and estimated by the number of cases in the selected studies. If the test is positive, the probability of actually being harmed is 96% (solid line in red). On the other hand, if the test is negative, the post-test probability of actually being harmed (blue dotted line) is close to 0.

## 4. Discussion

Since the most frequent dynamic of ankle trauma consists of the inversion mechanism, and since it firstly involves the ATFL, this ligament is the most frequently injured in all ankle traumas [2,45,46]. CFL injury may also occur in major inversion traumas, but it is almost always associated with an ATFL injury. The PTFL is rarely torn except in cases of complete ankle dislocation [3,47]. The most common mechanism of injury is inversion stress. MRI signs of ligamentous lesion include signal attenuation, laxity or discontinuity [4,48]. Ankle sprains are typically classified into three grades on both clinical and radiological criteria; ATFL rupture defines a Grade III injury [46]. This classification naturally affects the management of ankle sprains, hence the importance of a precise and reliable diagnosis [49,50,51].

Many imaging techniques have been employed to demonstrate ligamentous injuries of the ankle; these include Stress Radiography, which is effective but has limited diagnostic performance in the setting of an acute trauma due to patient pain and functional limitations [25]. Furthermore, US has proven to be useful in diagnosing ATFL injuries, with good sensitivity and specificity, wide availability in emergency departments and high cost-effectiveness [10]. While several studies evaluated MRI diagnostic accuracy for ATFL lesions in the setting of chronic ankle instability, a relatively small number of studies addressed the same problem in the setting of acute ankle trauma; to our knowledge, this is the first systematic review on this topic.

As our pooled data suggest, MRI shows excellent sensitivity and specificity in detecting ATFL injuries in the acute setting. On the one hand, these results are not surprising, as MRI is the most accurate imaging technique in evaluating ligamentous structures [22,46,52,53]. Moreover, our pooled sensitivity and specificity values are even better than those reported in the systematic review of Cao et al. [52], which conducted a similar analysis in the setting of chronic ankle instability (reporting pooled sensitivity of 0.83 and pooled specificity of 0.79). This observation suggests a possible better diagnostic performance of MRI in the acute trauma setting compared to chronic ankle instability, and needs to be further investigated. From a technical point of view, in the majority of the included studies MRI was performed using a 1.5 T magnet. Only in one study [43] was MRI performed with a 3T magnet; in another [38], both 0.5 T and 1.5 T magnets were used. Considering this small variability, a specific analysis of the diagnostic accuracy of acute ATFL lesions with varying MRI field strength was not carried out. Furthermore, it is reported that there are no significant differences in the diagnostic performance of MRI using a 3 T magnet rather than a 1.5 T one; significant advantages can only be obtained with a field strength of 7 T, which provides better depiction of ankle anatomy, fluid depositions, and cartilage defects [54,55,56,57]. All the selected studies except for Wei Tan et al. described the MRI protocols used, which showed substantial homogeneity: T2-weighted Turbo Spin Echo acquired in the axial plane was employed as the main sequence for a better visualization of the ATFL, using a section thickness of 3–4 mm with an interslice gap of 10%, with the only exception being Verhaven et al., in which a 3D-FSP sequence with a section thickness of 1 mm was employed. The oblique coronal-axial plane has already been indicated as the best one for visualizing the ATFL in previous studies [56,57]. None of the selected studies mentioned this scanning plane, but, as stated by Kim et al. [20], despite the possibility that the oblique axial-coronal plane could be added to routine MRI scanning protocol for better diagnosis of ATFL injury, the routine axial plane is still adequate and also allows a good evaluation of other ligamentous structures [57].

Four of the included studies provided data regarding MRI diagnostic performance in detecting both partial and complete ATFL lesions [37,41,42,44]. Three of them [38,39,40] provided data regarding complete lesions only. Wei Tan et al. [43] was the only study to report separate data for partial and complete injuries, in addition to global data. Due to the limited data available, separated pooled data for the two groups of lesions were not calculated; however, this could be an interesting topic for future studies.

Although our results may encourage the utilization of MRI in the setting of an acute ankle sprain, we believe that their reliability may be limited by some bias. For example, great heterogeneity was present among the included studies in terms of timing of MRI after the traumatic event; further research is needed to identify any differences in the diagnostic performance of MRI as its timing varies. Furthermore, while some of the studies [39,40,41] did not report precise selection criteria for patients operated on and/or undergoing arthroscopy, others considered for these procedures only patients with particularly severe clinical pictures [37,43] or with other clinical or instrumental findings suggestive of ATFL lesions [38,42,44], such as Talar Tilt > 15° on stress X rays or a positive Drawer test on physical examination. This may have biased our results, since MRI was performed on a patient population with a high pretest probability of ATFL injury. It would be of interest to assess the diagnostic accuracy of MRI on patients with less severe clinical features after ankle injuries. Strictly connected to this problem is the need to define precise indications for the use of MRI in the acute setting in light of its diagnostic potential. At present ankle injuries in an acute context are typically evaluated based on history (injury situation and mechanism, previous joint injury, etc.) and physical examination (e.g., inspection, palpation, loading status, testing special), while instrumental investigations should be required based on the indications proposed by the Ottawa Ankle Rules [58]. For ankle sprains that remain symptomatic for more than six weeks, Wolfe et al. [50] recommend that computed tomography (CT) or magnetic resonance imaging (MRI) should be considered. Therefore, in clinical practice, MRI is not routinely used in the hours or days immediately following acute ankle trauma, while it retains a fundamental role in surgical planning and in evaluating specific tissue pathologies, discerning between a very narrow range of differential diagnoses. On the one hand, in recent years, there has been an increasing interest in using MRI as a first-line tool [51] and our results might encourage this trend. On the other hand, major limitations to the use of ankle MRI as a first-line exam lie in its cost and in the fact that a large number of asymptomatic patients seem to have signal abnormalities of ATFL on MRI, as reported by Guillo et al. [53] and Stiell et al. [58], which could lead to an overestimation of ATFL injuries and trigger an expensive cascade of diagnostic tests or even inappropriate operative interventions [52,59]. This review was conducted by adopting strict inclusion and exclusion criteria in order to select only patients without a history of chronic ankle instability and with a correlation with surgery and/or arthroscopy, in order to obtain the most absolute possible MRI accuracy results. In clinical practice, this cannot always be faithfully reproduced, and therefore a future development could be to compare the accuracy of MRI in a group of patients like ours with another group with confounding factors such as chronic ankle instability or previous history of ankle surgery. To assess the effective utility of MRI in suspected ATFL lesions in the acute setting, it would be necessary to carry out studies that verify the impact of an early MRI diagnosis of these lesions on their prognosis and on patients’ functional recovery. Future analyses should be directed towards this goal, as well as towards investigating how therapeutic strategies can be influenced by the acute use of other methods such as ultrasound compared to MRI. Furthermore, reviews similar to this one could be useful to investigate the usefulness of MRI in trauma involving other musculoskeletal areas, such as scaphoid fractures and osteochondral knee injuries, as well as other clinical scenarios in which a delayed or misdiagnosis can have significant consequences with late complications [60,61].

## 5. Conclusions

This systematic review with meta-analysis studied the accuracy of MRI imaging for the diagnosis of acute ATFL injury, with the aim of assessing the clinical usefulness of an early diagnosis. Our results demonstrated that MRI shows high diagnostic accuracy in the diagnosis of acute ATFL lesions, presenting a sensitivity and diagnostic specificity around 1.0 (95% CI: 0.58–1) and 0.9 (95% CI: 0.79–0.96) respectively. These results suggest that routine MRI in the case of suspected ATFL acute injury may be clinically useful, although this is not done in clinical practice due probably to high cost. Therefore, further comparative systematic reviews with stress radiography and ultrasound may be useful in identifying the cases in which an in-depth diagnostic study with MRI is appropriate.

## Figures and Tables

**Table 1:** Detailed search strategies.

Step	MEDLINE	SCOPUS	CENTRAL
1	Accuracy [Title/Abstract] OR accurate rate [Title/Abstract] OR diagnostic value [Title/Abstract]	Accuracy [Title/Abstract]	MeSH descriptor: [Sensitivity and Specificity] explode all trees
2	Sensibitivity and Specificity [MeSH term]	Sensibitivity and Specificity [MeSH term]	accuracy: ti, ab, kw or accurate rate: ti, ab, kw or diagnostic value: ti, ab, kw (Word variations have been searched)
3	Acute ankle injury OR ankle trauma	Acute ankle injury	MeSH descriptor: [Lateral ligament, Ankle] explode all trees
4	ATFL	ATFL	ATFL (Word variations have been searched)
5	Talofibular [Text Word] OR anterior talofibular [Text Word]	anterior talofibular [Text Word]	acute ankle injury OR ankle trauma (Word variations have been searched)
6	Lateral ligament [MeSH terms]	Lateral ligament [MeSH terms]	Talofibular OR anterior talofibular (Word variations have been searched)
7	(1 OR 2) AND (3 OR 4 OR 5 OR 6)	(1 OR 2) AND (3 OR 4 OR 5 OR 6)	(1 OR 2) AND (3 OR 4 OR 5 OR 6)

**Table 2:** Summary of the included studies.

First Author (Year)	Subject Features	Age (Range)	Gender	Time from Injury to MRI Assessment	Gold Standard	MRI Protocol
Basha (2020)	29 patients with positive clinical findings suggestive of ATFL disruption	33 (23–43)	19 men and 10 women	Less than 3 weeks	Arthroscopy	1,5 T; axial T2-weighted images (T2), proton density
Gaebler (1997)	112 patients with inversion trauma to the ankle and typical clinical symptoms of a ligament injury	26 (16–35)	67 men and 45 women	Less than 5 days	Surgery	(PD), PD fat sat (PDFS), and short tau inversion recovery (STIR) sequences
Kreitner (1998)	18 patients with ankle injury	27 (9–42)	10 men and 8 women	Less than 7 days	Surgery	1,5 Tesla; two proton-density- and T2-weighted double spin-echo (SE), or two T2-weighted turbo-spin-echo (TSE) sequences in oblique axial planes followed by a 3D acquisition in the axial plane. Short tan inversion recovery (STIR) sequences in the coronal plane were performed in only a few cases
Oae (2003)	58 patients with ankle sprains and distal fibular fractures	37 (12–79)	32 men and 26 women	Not known	Arthroscopy and surgery	1,5 Tesla; transverse T1-weighted spin-echo and T2-weighted fast spin-echo sequences.
Oae (2009)	19 patients who needed an operation because of severe problems such as osteochondral lesions, synovitis and instability after acute ATFL injury	29 (13–55)	Not known	Less than 7 days	Arthroscopy and surgery	1,5 Tesla; transverse T2-weighted fast-spin-echo sequences
Takao (2003)	52 patients with acute injuries of the ankle	35 (14–67)	31 men and 21 women	Not known	Arthroscopy	1,5 Tesla; transverse T1-weighted spin-echo sequences and transverse T2-weighted fast spin-echo sequences
Wei Tan (2016)	40 patients with history of acute ankle sprain injury	26 (17–48)	35 men and 5 women	Less than 3 months	Surgery	3,0 Tesla; no other information about protocol
Verhavern (1991)	18 patients with an acute varus trauma of the ankle	21 (range not known)	Not known	Less than 6 hours	Surgery	1,5 Tesla; coronal and sagittal plane in 3D FISP (fast imaging with steady-state free precession)

**Table 3:** Methodological quality assessment of included study using QUADAS2 tool.

STUDY	RISK OF BIAS				APPLICABILITY CONCERN		
	PATIENT SELECTION	INDEX TEST	REFERENCE STANDARD	FLOW AND TIMING	PATIENT SELECTION	INDEX TEST	REFERENCE STANDARD
**Basha 2020**	+	+	+	+	+	+	+
**Gaebler 1997**	-	+	+	+	?	+	+
**Kreitner 1998**	?	+	+	+	?	+	+
**Oae 2003**	+	+	?	+	+	+	?
**Oae 2009**	+	+	+	+	+	+	+
**Takao 2003**	+	+	+	?	+	?	+
**Tan 2017**	+	-	+	+	+	+	+
**Verhaven 1991**	+	+	?	+	+	+	+

Red stands for high risk; Green stands for low risk; Yellow stands for risk unclear.

**Table 4:** Summary characteristic of the study.

First Author (Year)	TP	TN	FP	FN	Se	95% CI	Sp	95% CI
Basha (2020)	24	1	0	4	1.00	0.79–1.00	0.50	0.01–0.99
Gaebler (1997)	99	0	0	13	0.88	0.64–0.99	0.74	0.52–0.90
Kreitner (1998) *	18	0	0	0				
Oae (2003)	28	2	0	28	1.00	0.85–1.00	0.93	0.77–0.99
Oae (2009)	18	0	0	1	1.00	0.81–1.00	1.00	0.03–1.00
Takao (2003)	23	2	0	27	1.00	0.88–1.00	0.93	0.78–0.99
Wei Tan (2016)	15	6	2	17	1.00	0.96–1.00	1.00	0.75–1.00
Verhavern (1991)	16	1	0	1	1.00	0.86–1.00	0.80	0.28–0.99

Legend: Se = sensitivity; Sp = specificity; TP = true positive; FN = false negative. * = not included in the analysis.

## Data Availability

All data generated or analyzed during this study are included in the published article.
